# Neurofibromatosis in Children: Actually and Perspectives

**DOI:** 10.3390/children9010040

**Published:** 2022-01-02

**Authors:** Maria Lucia Sur, Ionel Armat, Genel Sur, Diana-Cristina Pop, Gabriel Samasca, Iulia Lupan, Teodora-Larisa Timis, Ioan-Alexandru Florian, Daniel Sur

**Affiliations:** 1 Department of Pediatrics, Iuliu Hatieganu University of Medicine and Pharmacy, 400006 Cluj-Napoca, Romania; surlucia@gmail.com (M.L.S.); diana.pop97@yahoo.com (D.-C.P.); dr.geni@yahoo.co.uk (D.S.); 2 Children Emergency Clinical Hospital, 400006 Cluj-Napoca, Romania; surgenel@yahoo.com; 3 Cardiovascular and Transplant Emergency Institute of Târgu Mureș, 540136 Targu Mures, Romania; armat_95ionel@yahoo.com; 4 Department of Molecular Biology, Babes-Bolyai University, 400084 Cluj-Napoca, Romania; lupan.iulia@gmail.com; 5 Department of Physiology, Iuliu Hatieganu University of Medicine and Pharmacy, 400006 Cluj-Napoca, Romania; doratimis@gmail.com; 6 Department of Neurosurgery, Iuliu Hatieganu University of Medicine and Pharmacy, 400006 Cluj-Napoca, Romania; florian.ioan.alexandru@gmail.com; 7 Institute of Oncology “Ion Chiricuta”, 400015 Cluj-Napoca, Romania

**Keywords:** malignant tumors, neurofibromatosis, children

## Abstract

The three types of neurofibromatosis, namely type 1, type 2, and schwannomatosis, are generally associated with various benign tumors affecting the skin and the nervous system. On rare occasions, especially in patients with neurofibromatosis type 1 (NF1), malignant neoplasms may also be present, several of them possessing a more aggressive course than in individuals without this syndrome. As such, a clear delineation between the three variants of neurofibromatosis is crucial to establish the correct diagnosis and management, as well as predict the neoplasm-related outcomes. Neurofibromin, the principal product of the NF1 gene, is a potent inhibitor of cellular proliferation, having been linked to several key signaling pathways involved in tumor growth. Therefore, it may provide a useful therapeutic target for tumor management in these patients. In this article, we want to present the association between deficiency of neurofibromin and the consequences of the lack of this protein leading to different kinds of malignant tumors. The therapy is still uncertain and most therapeutic options are in development or clinical trials.

## 1. Introduction

Neurofibromatosis denotes a spectrum of multisystem genetic disorders mainly characterized by benign neurocutaneous neoplasms [1]. The most predominant variants of this disease are neurofibromatosis type 1 (NF1), accounting for as many as 96% of cases, and type 2 (NF2), which amounts to 3% of patients respectively. Schwannomatosis (SWN) also belongs to this pathological field and has overlapping diagnostic features with NF2, however, its occurrence is rarer and more poorly understood [1,2,3]. Despite being inherited via an autosomal dominant pattern, these entities possess distinct genetic origins. NF1 symptoms usually occur during early childhood.

Malignant tumors in neurofibromatosis are infrequent, among which malignant peripheral nerve sheath tumors (MPNST) arise during the lifetime of up to 15% of patients with NF1 [4]. Around 50% of MPNSTs occur in individuals with NF1 [5]. Chronic lymphocytic leukemia and diffuse B cell lymphomas may also occur in these patients, although these associations are exceptionally rare [6,7]. Other cancers more commonly encountered in patients with NF1 include the malignant triton tumors (MTTs), which are a rare variant of MPNSTs [8,9,10,11]. The malignant transformation of spinal low-grade astrocytomas and the appearance of cerebral glioblastomas associated with NF-1 have also been documented [12,13]. Moreover, a recent cohort study established that patients with NF1 not only possessed significantly lower disease-specific survival (DSS) ratios for undifferentiated pleomorphic sarcomas, high-grade gliomas, MPNST, melanomas, or ovarian carcinomas in comparison to other tumors but also a lower age of occurrence for these neoplasms than in individuals without NF1 [14]. The epidemiology and natural history of these associations remain unelucidated in pediatric patients. The purpose of this review is to shed light on the correlation between neurofibromatosis and reported malignancies, with a focus on NF1 and its characteristically defective protein neurofibromin, as well as establish potential directions of research.

## 2. The Variants of Neurofibromatosis

The three forms of neurofibromatosis, namely NF1, NF2, and SWN are caused by distinct mutations in specific genes. They are all associated with the occurrence of multiple tumors and malformations concerning several parts of the body [15]. The tumors differ significantly between NF1 and the latter two variants, whereas both NF2 and SWN have multiple schwannomas and meningiomas as defining features. The following paragraphs present general individual characteristics of each variant, whereas clinical manifestations and diagnostic criteria are analyzed comparatively in ulterior sections.

### 2.1. Neurofibromatosis Type 1

Neurofibromatosis type 1 (NF1) or von Recklinghausen disease is an autosomal-dominant genetic disease, caused by a mutation of NF1, localized on chromosome 17. The mutations can vary widely, like translocations, deletions, inversions, and point mutations. Around half of all causative mutations appear de novo [16]. This altered gene is tasked with encoding neurofibromin, which is a tumor-suppressive protein that ordinarily regulates cell growth [17,18]. The prevalence of NF1 is approximately 1 in every 3500 live births [18,19] and can be easily diagnosed via its clinical features consisting of cafe au lait spots, axillary freckles, Lisch nodules, or neurofibromas [15,20].

Neurofibromin deficiency is typically associated with tumors arising in various sites of the human body. Patients with this condition present a higher risk of developing tumors than the general population; this risk is estimated at 60% [18]. Nervous system tumors are commonly associated with the three types of neurofibromatosis, out of which gliomas are most frequent in NF1. Optic gliomas are presented in 15% to 20% of cases [18,21,22].

The pediatric population with NF1 gene defects includes patients at risk for developing hematopoietic malignancies such as acute or chronic leukemias or different types of lymphomas [17,23]. Additionally, patients may also develop tumors that involve the endocrine system, like pheochromocytomas or multiple gastrointestinal stromal tumors (GIST). Melanomas can also occur, as can breast cancer in adult women [24,25].

### 2.2. Neurofibromatosis Type 2

Type 2 neurofibromatosis (NF2) has a prevalence of 1 in 33,000 births, without a predilection for a certain race or gender [26]. Affected patients typically present around 20 years of age. Its primary features include bilateral vestibular schwannomas, as well as meningiomas that can vary in location and number [1]. The NF2 gene, which leads to the occurrence of this disease, is located on chromosome 22 and produces a protein called *merlin* (also known as *schwannomin*), a tumor suppressor. *Merlin* has been shown to act on the *PI3kinase/Akt, Raf/MEK/ERK*, and *mTOR* pathways [26]. Mutations of these genes are most frequently truncating and lead to severe forms of NF2, yet single and multiple exon deletions are also common. These result in a loss of merlin, leading to unfettered cell growth [18]. The outcome is negatively affected by the early age of the patient at disease onset, a higher number of meningiomas, and the presence of a truncating mutation. It may be argued that neurofibromatosis is a misnomer for this specific syndrome since neurofibromas are relatively rare in NF2 [27].

### 2.3. Schwannomatosis

Schwannomatosis (SWN) is a syndrome distinguished by a concomitant development of multiple peripheral nerve schwannomas in the absence of vestibular nerve involvement [27]. Despite the significant phenotypical overlap between SWN and NF2, especially considering that schwannomas are the predominant tumors in both diseases; SWN is conventionally regarded as the third form of neurofibromatosis. This variant has an incidence of 1 in 60,000 births [28]. To date, two distinct genes are known to cause SWN, both different from those involved in the previous forms. Mutations in the *SMARCB1* (otherwise known as *INI1*, *BAF47*, or *hSNF5* gene and centromeric to the NF2 gene situated on chromosome 22) and *LZTR1* genes (also located on chromosome 22, close to the former gene), which suppress tumors, are associated with this type of neurofibromatosis [16,18]. The majority of mutations occur sporadically, however, an autosomal-dominant inheritance pattern has also been observed in familial cases [3,27,29]. Interestingly, mutations of *SMARCB1* have also been incriminated for the development of rhabdoid tumors in the rhabdoid predisposition syndrome; although these syndromes have scant overlapping features [27].

### 2.4. Legius Syndrome

Legius syndrome is a condition characterized by changes in skin coloring pigmentation. Almost all affected individuals have multiple café-au-lait spots, and freckles in the armpits and groin may occur in some affected individuals. Many of the signs and symptoms of Legius syndrome also occur in NF1. It can be difficult to tell the two disorders apart in early childhood. However, the features of the two disorders differ later in life.

## 3. Clinical Manifestations

NF1 has several particularities according to age, but the most important characteristics are the cafe au lait spots, Lisch nodules, and axillary or inguinal freckles, which are also part of the diagnostic criteria [30]. Neurofibromas are the defining neoplastic manifestation of NF1, being either cutaneous (pedunculated, nodular, or plaque-like) or belonging to the deep soft tissues [27]. Patients with NF1 also harbor a propensity to develop gliomas, both low- and high-grade, the most common of which involve the optic pathway. The brainstem may also be affected by gliomas, these representing the second most frequent type of gliomas in NF1. Malignant tumors are rare and not featured within the diagnostic criteria, being discussed in a later section. Table 1 reviews the clinical features respective of each variant of neurofibromatosis occurring sequentially according to the age of the patient. NF1 clinical manifestations are frequently associated with pain. A study conducted by Alanna M Kongkriangkai et al. showed that pain may occur at a young age, 41% of the participants requiring medication to manage these symptoms [26]. The same study presented a significant difference between children and adults regarding the source of pain, with migraine being the most reported in children and NF tumors in adults. Cognitive deficits including significant impairments in learning, attention, and behavior are found in about 70% of children with neurofibromatosis [21].

NF2 patients develop nervous system tumors, most commonly schwannomas (vestibular or of other cranial or peripheral nerves) but also meningiomas and ependymomas [27]. The schwannomas generally occur before the age of 30 years, even as early as during childhood. Ocular features may also be present in as many as 80% of patients, particularly cataracts arising from posterior subcapsular lenticular opacities [31]. Other possible ocular manifestations are epiretinal membranes, hamartomas of the retina, intraocular schwannomas, as well as gliomas and meningiomas involving the optic nerve.

For SWN, the peak age of presentation is during adulthood, somewhere between 30 and 60 years of age, reportedly later than for NF2 [3,32]. Additionally, SWN patients have an improved life expectancy than those suffering from NF2. The major symptom is chronic debilitating pain caused by the compression of schwannomas on the nerves. The tumors tend to possess an intraneural growth pattern, present peritumoral edema, myxoid change, as well as mosaic INI1 staining on immunohistochemistry studies.

## 4. Diagnosis

The diagnosis needs predominantly clinical criteria, which have been recently revised by an international consensus [33]. For a positive diagnosis of NF1, a minimum of two out of the seven criteria mentioned in Table 2 and Table 3 are required for patients whose parents are not affected by NF1, or just one in the case where one of the parents has been diagnosed with this disease [33,34].

For NF2, however, the cutaneous features are more subtle and less common than in NF1 [35]. Bilateral vestibular schwannomas are pathognomonic for NF1, although many of the patients (around 40%) do not present these tumors on both sides at the time of initial investigations [27]. The cutaneous finding most frequently described is an elevated plaque-like lesion that might present hyperpigmentation compared to the surrounding skin. Additional skin abnormalities are represented by the subcutaneous nodules denoting swelling of the nerves, as well as cutaneous tumors that typically represent schwannomas rather than neurofibromas. Furthermore, a definitive diagnosis can be established if there are positive clinical criteria and a confirmed affected first-degree relative. In the absence of these features, a genetic variant may be required to establish the mutation in one of the respective NF genes. As of yet, the diagnostic criteria of SWN have not been as stringently standardized as those for the more common variants of neurofibromatosis. Table 3 illustrates the diagnostic criteria for each of the three variants of neurofibromatosis.

Diagnosis criteria for Legius Syndrome are presented in Table 3.

## 5. The Action Mechanism of Neurofibromin

Neurofibromin is the product encoded by the NF1 gene, (*17q11.2*). This protein acts as a suppressor against the proliferation of various cell types. The gene in question can undergo a large variety of mutations, however, only a small fraction of these mutations have a confirmed correlation between genotype and phenotype. For example, the microdeletion of this gene is associated with a severe form of the syndrome [36,37].

*Neurofibromin* levels fluctuate in different tissues following the developmental and differentiation stages of the organism. It regulates cell proliferation and differentiation through several signaling pathways, such as the Ras pathway. As such, *neurofibromin* is a *GTPase-activating protein* (GAP) and functions as a negative regulator of the Ras pathway activity, which starts with the activation of the growth factor receptor (*GFR*). *GFR* causes the transformation of *RAS-GDP* protein from inactive to its active form. *Neurofibromin* acts as a modulator of this activation, by triggering intrinsic *GTPases*, leading to downregulation of *RAS* [16,18].

The *RAS* gene family comprises several *GTP*-coupled genes with a well-known role in proliferation and differentiation. These are *KRAS*, *HRAS*, *NRAS*, which are also regulated by other genes such as *SHOC* and *SPRED1*. Mutations that target this gene can lead to Legius syndrome. The *RAS* gene family further controls the *BRAF* / *RAF1* pathway, and through the *BRAF* pathway, the *MAP2K1* and *MAP2K2* pathways, *ERK 1* and *2*, ultimately leading to multiple control targets located in the nucleus and cytoplasm that allow control of proliferation, maturation, and differentiation processes and cellular apoptosis. Mutations that affect various proteins in the control of these signaling pathways can lead to various pathologies, the most widely known being the Noonan syndrome, Noonan syndrome with multiple lentigines, also referred to as LEOPARD, Legius syndrome, and Cardio-facial-cutaneous syndrome. These syndromes are encompassed by the so-called RASopathies [18].

Aside from *RAS*, *neurofibromin* also plays an important role as a tumor regulator and suppressor via other signaling pathways: the *tyrosine kinase receptor*, the *endothelin* receptor B, the *tyrosine kinase* anaplastic lymphoma *kinase receptor*, and the *granulocyte-macrophage* colony-stimulating factor receptor (*GM-CSF*). The most important are the *RAS* or *mitogen-activated protein kinase* (*MAPK*) and *phosphoinositide 3-kinase* (*PI3K*) or mechanistic target of rapamycin (*mTOR*) pathways [18,36,38].

## 6. Types of Tumors

### 6.1. Neurofibromas

As the name of the disease implies, the patients with neurofibromatosis develop multiple neurofibromas, which are benign tumors. They usually appear in early childhood and adolescence. In the majority of cases, patients develop subcutaneous or plexiform neurofibromas (PN), which can transform into malignant variants that are associated with the invasion of underlying structures [39]. This aggressive form of sarcoma, resistant to multiple courses of chemotherapy, is known as malignant peripheral nerve sheath tumors (*MPNSTs*) and has a severe prognosis [18,40]. These tumors possess a high probability of recurrence after surgical resection and can also metastasize early, representing the main cause of lowered life expectancy in these patients. Altered mechanisms of proliferation affected by NF gene mutation attract the proliferation of a multitude of cells: macrophages, fibroblasts, mast cells inside the nerve. PNs may transform into *MPNSTs* in 10% of cases due to the additional accumulation of mutations in other tumor suppressor genes, such as *p53*, *SUZ12*, *EED*, the NF1 gene mutation being the initial trigger [18,41,42].

MTTs represent a rare subtype of *MPNSTs* that upon inspection are sturdy, large, grayish tan neoplasms with intrinsic zones of hemorrhage and necrosis [8,9,10,11]. They are very rare, since *MPNSTs* comprise between 5% and 10% of all soft tissue sarcomas, while *MTTs* represent less than 10% of all *MPSNTs*. They are mostly associated with young NF1 patients and have extremely aggressive behavior.

Neurofibromas located at the intestinal level are referred to as gastrointestinal stromal tumors (GIST). The main site of development is the small intestine, with the tumors being characteristically multiple. The NF1-associated GIST harbor different mutations than those that appear de novo. It is believed that the appearance of NF1-associated GIST requires several distinct mutations to appear, aside from the second hit of the NF1 gene [22,24].

### 6.2. Gliomas

The patients with mutations in either of the NF genes may develop a wide variety of central and peripheral nervous system tumors, such as low- and high-grade astrocytomas, meningiomas, or schwannomas of cranial nerves, the last two being more frequently associated with NF2 deficiency. Gliomas of the optic pathways and brainstem usually occur during childhood and are linked to NF1 gene mutations [43,44]. Optic nerve gliomas are the more common type encountered in children, seen in 15% to 20% of individuals with this condition [43], denoting a low-grade pilocytic astrocytoma that may rarely suffer from malignant transformation [45]. These gliomas might arise at any part of the optic pathway, most commonly at the optic nerves themselves as well as the optic chiasm. Owing to their location, about 35–50% of children affected by optic gliomas present with reduced visual acuity [46].

The second site typically affected is the brainstem, yet patients with gliomas at this location tend to be older. These tumors may develop at any segment of this structure (medulla, pons, or midbrain). Generally, brainstem gliomas in patients with NF1 deficiency are asymptomatic and discovered incidentally in magnetic resonance imaging (MRI) for unrelated complaints. However, a small number of these patients can be symptomatic, yet with nonspecific symptoms such as headache, nausea, and vomiting [43]. While children with NF1 generally harbor low-grade gliomas, which are considered less aggressive, adults with the same syndrome may develop malignant gliomas. The incidence of these tumors in adults is much lower than in children.

Ependymomas are a type of low-grade glial tumors primarily found within the ventricles or the ependymal canal, arising from the ependymal cells lining these structures [47]. They are more commonly found in the spinal cord of patients with NF2 than in individuals without this syndrome. The cervical and cervicospinal junction represent the most frequent sites, the tumors generally being multiple and with a ‘string of pearls’ appearance [48]. Surgery may help alleviate symptoms; however, there is a great risk of neurological deterioration [49]. For those that are asymptomatic, repeat imaging monitoring remains the mainstay of management.

### 6.3. Schwannomas

Schwannomas of the vestibular branch of the eighth cranial nerves are the most common in patients with NF2 gene mutations, although these neoplasms may also arise on any other cranial nerve. They are benign tumors comprised of spindle cells with combined Antoni A and Antoni B cellular arrangements, Verocay bodies, as well as hyalinised vessels [27]. The tumors themselves are well-delineated, encapsulated, and usually lead to a displacement and distortion of the nerve fibers instead of engagement [34]. Initial symptoms include dizziness, balance difficulties, tinnitus, and even hearing loss or facial nerve involvement in later stages [26]. Patients require periodic hearing evaluation, repeated MRI scans, and brainstem evoked potentials to assess tumor progression. Neurosurgical removal remains the first line of therapy, yet NF2 schwannomas present a higher recurrence rate than in individuals without the disease. Radiation may be useful, yet it bears the risk of malignant transformation. Auditory brainstem implants are a valuable option in cases where the cochlear nerve must be sacrificed [50]. Bevacizumab is a vascular endothelial growth factor (VEGF) inhibitor and has been shown as effective in reducing tumor size and improving hearing in nearly one-third of treated patients [51].

### 6.4. Meningiomas

In descending order of frequency, the second type of tumor in patients with NF2 gene mutations are meningiomas, the intracranial location being more common and with earlier onset than those within the spine. They are associated with substantial morbidity and mortality in this population [27]. Intriguingly, between 20% and 40% of pediatric patients with meningiomas harbor the NF2 mutation [27,52], and up to 1% of all meningiomas are correlated with this syndrome [53]. As the name suggests, these tumors arise from the meningeal layers of the brain and spinal cord. Gross total surgical removal ensures a 90% 5-year progression-free survival rate, however, radiation therapy can also be provided for patients with incompletely removed or recurring meningiomas.

### 6.5. Lymphoproliferative Malignancies

A minority of children present a high risk for developing aggressive tumors, such as hematopoietic malignancies, especially myeloid leukemia. Several types of hematopoietic malignancies like acute and chronic leukemias, respectively lymphoblastic and myelomonocytic leukemias correlated with NF1 gene defects have been reported. Associations of non-Hodgkin’s lymphoma with NF1 have also been described [54]. However, a recent study by Bergqvist et al. suggested that the lymphoproliferative malignancies in NF1 patients may not possess a higher incidence than the general population of France [55].

### 6.6. Endocrine Tumors

Endocrine tumors are frequent in these patients like gastrointestinal stromal tumors, pheochromocytoma of the adrenal glands, and paragangliomas. These types of tumors may be hormone-secreting tumors such as adrenaline, noradrenaline, and dopamine, which lead to cardiovascular and neurological symptoms and non-secreting tumors, and are often found imagistically without having clinical manifestations, with a recommended annual screening of these types of tumors. Pheochromocytomas may be present from the beginning unilaterally, bilaterally, or they may be ectopic, some having a metastatic character [17,56].

### 6.7. Breast Cancer

It was observed that women under 40 years of age with NF1 have a higher risk of developing breast cancer than the general population [54]. Moreover, this type of cancer has been correlated with a poorer prognosis than in non-NF1 patients, as well as estrogen and progesterone receptor negativity and overexpression of HER2, demanding active yearly controls [57,58]. This mutation is also very common in different types of cancer, such as ovarian cancer, glioblastoma, and skin cancer. Therefore, this gene likely plays an important role in tumorigenesis [18,20,59].

### 6.8. Melanomas

It has also been established that the NF1 mutation affects the turnover of melanocytes and the distribution of melanin, which phenotypically translates into the appearance of cafe au lait spots and axillary and inguinal freckles. Cafe au lait spots, which are part of the defining criteria of the disease, are patches of skin hyperpigmentation explained by an increased number of keratinocytes reported to melanocytes. The synthesis of melanin is intensified upon the inactivation of the NF1 gene as a result of increased signaling activity via the PKA and Erk pathways, which are cAMP-mediated. Melanomas may be associated with neurofibromatosis due to the common origin of the neural crest for both melanocytes and Schwann cells [60,61]. As such, another type of tumor with a higher incidence of neurofibromatosis is represented by melanomas. A rare type of melanoma that may arise in NF1 patients is desmoplastic melanoma. The mutations of the NF1 gene were also observed in sporadic melanomas, but it is more common in melanomas after exposure of the skin to radiation, or wild-type melanomas, which include mutations in BRAF or Nras genes. Recently, it has been demonstrated that the NF1 gene mutation is merely a trigger for developing melanomas [60].

## 7. Treatment

Being a genetic, multisystemic disorder, neurofibromatosis requires a multi-disciplinary approach. One of the therapeutic and curative possibilities would be gene therapy, yet the lack of extensive experience represents a detracting factor. There is a possibility of completely replacing the mutant gene or only partially substituting it with satisfactory levels of functional neurofibromin [62].

Currently, genome editing techniques are considered more feasible, leading to a permanent correction of gene mutations. Pathophysiologically, the disease is characterized by a mutation within the NF1 gene that leads to the absence of neurofibromin, which has a tumor suppressor role, leading to the increased RAS activity and subsequent oncogenic potential [63]. Therefore, the RAS gene itself or its products may represent potential therapeutic targets in neurofibromatosis via two possible directions: either direct inhibitors of the RAS gene or inhibitors of the products resulting from the modulation pathways of RAS [64,65].

RAS inhibitors appeared due to the lack of binding sites with pharmacological potential at the RAS surface. Molecules with inhibitory potential on RAS are obtained by prenylation, using a farnesyltransferase that catalyzes the prenylation of the RAS gene. Attempts have been made to introduce and clinically test this type of molecule, Tipifarnib, however, research is still needed [62,63,66].

On the other hand, molecules developed with the suppressive role of RAS pathway effectors include protein kinases that bind to the various sites of RAS binding. The first molecule of this type was Sorafenib, with no demonstrated beneficial results in clinical trials, yet it remained in testing for gliomas. Another molecule with therapeutic potential is Rigosertib, in testing for myelomonocytic leukemia, but with increased toxicity [62,67].

Molecules with therapeutic potential in clinical trials are Sirolimus and Everolimus, both inhibitors of the mTOR signaling pathway [63,68]. Sirolimus is an mTOR inhibitor with antiproliferative, and antitumoral effects acting on the mTOR/PI3K/AKT signaling pathway implicated in angiogenesis resulting in cellular growth and vascular proliferation [69]. Tyrosine kinase molecules (Imatinib) have been approved for clinical trials since in neurofibromatosis there is a wild-type RAS, different from that expressed in other types of cancers [69]. A recent study showed the benefit of selumetinib in children with PN. Most children included in this study had durable tumor shrinkage and clinical benefits [70]. For NF1-related cognitive deficits, the serotonin 5 hydroxytryptamine 6 (5-HT_6_) receptor is considered a potential therapeutic target [70]. It has been shown that the 5-HT_6_ receptor leads to an increase in cAMP formation, although whether this is linked to the neurofibromin-dependent cAMP production found in NF1 is currently unknown. Nevertheless, according to the study by Nadim et al., there is a physical interaction between neurofibromin and the 5-HT_6_ receptor, which may stand at the forefront of the neuronal abnormalities in NF1 patients [71].

## 8. Conclusions

The tumors differ significantly between NF1 and the latter two variants, whereas both NF2 and SWN have multiple schwannomas and meningiomas as defining features. Malignant tumors associated with NF1 are rare and not featured within the diagnostic criteria. The schwannomas occurring in NF2 vulnerable patients. The major symptom of SWN patients is chronic debilitating pain caused by the compression of schwannomas on the nerves. The diagnostic criteria of SWN have not been as stringently standardized as those for the more common variants of neurofibromatosis. Neurofibromin plays a role as a tumor regulator and suppressor via other signaling pathways. The therapeutic and curative possibilities are gene therapy. The RAS gene is the therapeutic target in neurofibromatosis.

## Figures and Tables

**Table 1:** Clinical manifestations for Neurofibromatosis type 1 (adapted after [14]).

Infancy	Early Childhood	Adolescence	Adulthood
Café au lait spots; Sphenoid bone dysplasia/tybial dysplasia, pseudoarthrosis; Plexiform neurofibroma	Motor/speech delays Optic gliomas; Autism spectrum disorder or attention deficit disorder Difficulties learning	Axilar/inguinal freckles; Lisch nodules Scoliosis Neurofibromas Brainstem glioma	Malignant peripheral nerve sheath tumors High-grade glioma Breast cancer

**Table 2:** Diagnostic criteria for the neurofibromatoses.

Type 1 Neurofibromatosis (NF1)	Type 2 Neurofibromatosis (NF2)	Schwannomatosis
(A) At least 2 out of 7 criteria should be met to establish a diagnosis in individuals without NF1 parents: ≥6 café-au-lait spots (>5 mm before puberty, >15 mm after puberty) * Axillary or inguinal freckling * ≥2 neurofibromas of any kind or ≥1 plexiform neurofibroma ≥2 Lisch nodules (iris hamartomas) seen via slit lamp examination, or ≥2 choroidal anomalies identified via OCT or NIR Optic nerve glioma Typical lesions of bones (dysplasia of sphenoid bone, pseudarthrosis of a long bone, or anterolateral tibial bowing) Heterozygous pathogenic NF1 variant with a fraction of 50% of the variant allele in seemingly healthy tissue or cells such as leukocytes (B) If the parent of a child meets the diagnostic criteria specified in A, the child in question merits a diagnosis of NF1 if they exhibit one or more of the criteria in A. Mentions: * If only café-au-lait spots and axial/inguinal freckling are present, NF1 is highly likely the diagnosis, however, Legius syndrome may be the actual diagnosis in exceptional cases. At least one of the two aforementioned pigmentary findings should be bilateral. ** Sphenoid wing dysplasia does not represent a distinct criterion in the case of an ipsilateral orbital plexiform neurofibroma.	Any one of the criteria below: Bilateral vestibular schwannomas (VS) in patients under 70 years Unilateral VS under 70 years and a first-degree relative with NF2 Any two of the following: meningioma, schwannoma (non-vestibular), neurofibroma, glioma, cerebral calcification, cataract AND first-degree relative with NF2 OR unilateral VS and negative *LZTR1* testing Multiple meningiomas and unilateral VS or any TWO of the following: schwannoma (non-vestibular), neurofibroma, glioma, cerebral calcification, cataract Constitutional or mosaic characteristic mutation of NF2 gene retrieved from blood samples, or identification of an identical mutation within two distinct tumors in one patient.	Any one of the criteria below: ≥2 non-intradermal anatomically distinct schwannomas (≥1 confirmed by histology) Cranial imaging study with no evidence of bilateral VS Absence of NF2 gene mutation ≥1 histologically confirmed schwannoma, unilateral VS or intracranial meningioma AND an affected first-degree relative with confirmed schwannomatosis A germline *SMARCB1* or *LZTR1* pathogenic variant AND ≥ 1 histologically confirmed schwannoma or meningioma Note: The presence of a unilateral VS or meningioma(s) does not exclude the diagnosis of schwannomatosis.
Genetic tests: blood analysis for constitutional mutations and tumor analysis (if tumor material is available, fixed, or frozen) for somatic mutations (mosaics). Diagnostic criteria for mosaic neurofibromatosis type 1 are: A pathogenic heterozygous NF1 variant with a variant allele fraction of significantly less than 50% in apparently normal tissue such as white blood cells AND one other NF1 diagnostic criterion (except a parent fulfilling diagnostic criteria for NF1), an identical pathogenic heterozygous NF1 variant in two anatomically independent affected tissues (in the absence of a pathogenic NF1 variant in unaffected tissue), A clear segmental distribution of café-au-lait macules or cutaneous neurofibromas and another NF1 diagnostic criterion (except a parent fulfilling diagnostic criteria for NF1), or a child fulfilling diagnostic criteria for NF1.		
NF1 gene analysis	NF2 gene analysis	NF2 gene analysis (negative), *SMARCB1 LZTR1* (at least 1 positive for germline mutation)

* The presence of fewer than six café-au-lait spots does not exclude Legius syndrome. ** Different tissues originating from the same primary affected lesion count for one tissue only. Table adapted after [3,27,33].

**Table 3:** Diagnostic criteria for Legius syndrome.

A: The diagnostic criteria for Legius syndrome are met in an individual who does not have a parent diagnosed with Legius syndrome if the following CRITERIA are present:	The diagnostic criteria for mosaic Legius syndrome are met in an individual if any of the following is present:
• Six or more café-au-lait macules bilaterally distributed and no other NF1-related diagnostic criteria except for axillary or inguinal freckling *	1. A heterozygous pathogenic *SPRED1* variant with a variant allele fraction of significantly less than 50% in apparently normal tissue such as white blood cells AND six or more café-au-lait macules
• A heterozygous pathogenic variant in *SPRED1* with a variant allele fraction of 50% in apparently normal tissue such as white blood cells	2. An identical pathogenic heterozygous *SPRED1* variant in two independent affected tissues (in the absence of a pathogenic *SPRED1* variant in unaffected tissue) **
B: A child of a parent who meets the diagnostic criteria specified in A merits a diagnosis of Legius syndrome if one or more of the criteria in A are present	3. A clear segmental distribution of café-au-lait macules AND a child fulfilling the criteria for Legius syndrome

* The presence of fewer than six café-au-lait spots does not exclude Legius syndrome. ** Different tissues originating from the same primary affected lesion count for one tissue only. Table adapted after [33].

## Data Availability

Not applicable.
